# SVR-EEMD: An Improved EEMD Method Based on Support Vector Regression Extension in PPG Signal Denoising

**DOI:** 10.1155/2019/5363712

**Published:** 2019-12-12

**Authors:** Guangda Liu, Xinlei Hu, Enhui Wang, Ge Zhou, Jing Cai, Shang Zhang

**Affiliations:** College of Instrumentation and Electrical Engineering, Jilin University, Changchun 130026, China

## Abstract

Photoplethysmography (PPG) has been widely used in noninvasive blood volume and blood flow detection since its first appearance. However, its noninvasiveness also makes the PPG signals vulnerable to noise interference and thus exhibits nonlinear and nonstationary characteristics, which have brought difficulties for the denoising of PPG signals. Ensemble empirical mode decomposition known as EEMD, which has made great progress in noise processing, is a noise-assisted nonlinear and nonstationary time series analysis method based on empirical mode decomposition (EMD). The EEMD method solves the “mode mixing” problem in EMD effectively, but it can do nothing about the “end effect,” another problem in the decomposition process. In response to this problem, an improved EEMD method based on support vector regression extension (SVR-EEMD) is proposed and verified by simulated data and real-world PPG data. Experiments show that the SVR-EEMD method can solve the “end effect” efficiently to get a better decomposition performance than the traditional EEMD method and bring more benefits to the noise processing of PPG signals.

## 1. Introduction

PPG [1] is a promising biometric technique based on Lambert–Beer's law [2] and the difference in spectral absorption characteristics of human skin and blood to convert optical signals into blood volume and blood flow information. It can be used for noninvasive detection of microvascular blood flow changes, providing quantities of possibilities in detecting blood volume and blood flow parameters [3–5]. Unfortunately, the noninvasiveness of PPG has both advantages and disadvantages: PPG signals are susceptible to disturbances from external environment and thus it causes inaccuracies to the measured results and those disturbances, including respiratory activities (RA), motion artifacts (MA), power line interference, and high-frequency noise generated by electronic components, tend to cause PPG signals to be doped with nonlinear and nonstationary components, which can result in spectral aliasing and distortion when processed with traditional methods.

The EMD method proposed by Huang et al. [6] in 1998 decomposes the time series into a set of intrinsic mode functions (IMFs), and noise can be eliminated by selecting appropriate IMFs. However, some drawbacks impede its further development. Several years later, a more powerful ensemble EMD [7] method called EEMD is presented and solves the “mode mixing” problem, one of the major drawbacks of the original EMD. The EEMD method has proven to be quite versatile in a broad range of applications such as geology [8, 9], banking [10], machinery [11, 12], and medicine [13] for extracting signals from data generated in noisy processes. Respecting the denoising of PPG signals, lots of researches have also been carried out. Sweeney et al. [14] used EEMD with canonical correlation analysis to remove artifacts both from electroencephalography (EEG) and functional near infrared spectroscopy (fNIRS) single channel data; Liao et al. [15] used the EEMD method to achieve accurate analysis for PPG signals and implemented it on a specific platform; Chuang et al. [16] analyzed the high-frequency band (0.4–0.9 Hz) of IMF^5th^ decomposed by EEMD to measure pulse rate variability (PRV); Motin et al. [17] proposed an algorithm based on EEMD with principal component analysis (EEMD-PCA) as a novel approach to estimate heart rate (HR) and respiratory rate (RR) simultaneously from PPG signals; Sadrawi et al. [18] used PPG data corrupted by vertical MA noise to evaluate the performance of EEMD filtering.

The EEMD method overcomes the “mode mixing” problem in EMD, but it does not consider the second problem existing at the same time: “end effect,” which causes the two ends of the time series to diverge when spline interpolation. In order to solve this problem, this paper proposes an improved EEMD method (SVR-EEMD) based on support vector regression extension and verifies its denoising performance by simulated data and real-world PPG data.

This paper will first describe the experimental materials and introduce the principle of the SVR-EEMD method and its implementation steps. Then, we will report the results of the proposed method on the simulated data and real-world PPG data and compare the denoising performance of different methods, and further advices on the necessary research are also discussed. Finally, we will draw the conclusion part to clarify the effectiveness and efficiency of this method.

## 2. Materials and Methods

### 2.1. Simulated Data Acquisition

The simulated signal which is sampled at 1 kHz for a duration of one second consists of a sinusoidal signal of 5 Hz and a cosine signal of 20 Hz. It can be expressed by equation (1), where *n*(*t*) is the superimposed Gaussian white noise to ensure that the signal-to-noise ratio (SNR) of the simulated signal is 15 dB:

(1) $$\begin{array}{c} y\left(t\right)=\sin\left(2*\pi *5*t\right)+\cos\left(2*\pi *20*t\right)+n\left(t\right). \end{array}$$

The SNR is calculated by equation (2), in which *s*(*t*) is the signal component that is equal to the first two parts on the right-hand side of equation (1) and *n*(*t*) is the noise component. Accordingly, we can calculate the noise intensity which is 0.0316 in the simulated signal:

(2) $$\begin{array}{c} \text{SNR}=10*\log\frac{\sum s{\left(t\right)}^{2}}{\sum n{\left(t\right)}^{2}}. \end{array}$$

### 2.2. Real-World PPG Data Acquisition

The real-world PPG data are obtained from BIDMC PPG and Respiration Dataset of PhysioBank, which is supported by the National Institute of Medical Sciences (NIGMS) and National Institute of Biomedical Imaging and Bioengineering (NIBIB) and whose data were originally acquired from critically ill patients during hospital care at the Beth Israel Deaconess Medical Centre (Boston, MA, USA) [19, 20]. There are a total of 53 sets of patient data in the dataset, each of which records some basic information of the patient and a series of physiological data of certain duration. These physiological data include respiratory activity data, EEG data, PPG data, and so on. We picked 10 sets (2, 5, 33, 34, 37, 38, 43, 45, 50, and 53) of PPG data to carry out the real-world PPG data experiment of this study.

### 2.3. The Proposed SVR-EEMD Method

**Figure 1:**
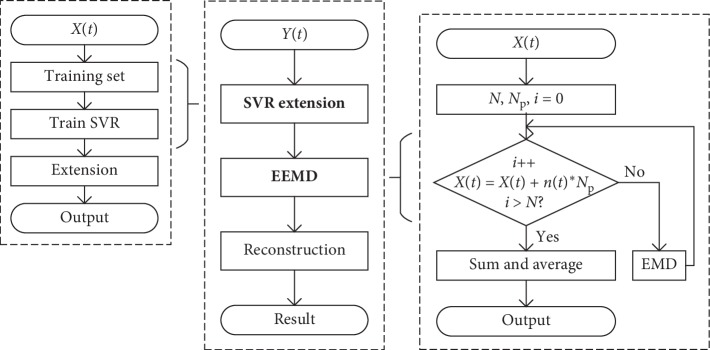
Implementation process of the proposed SVR-EEMD method. The left part describes the signal extension procedure, and the right part describes the signal decomposition procedure.

The SVR-EEMD method can be generally implemented by two steps: firstly, construct a training set based on the original signal to train the SVR model and use the trained SVR model to extend a finite number of maxima and minima time series, respectively, to the left and right ends of the original signal; then the EEMD algorithm is performed on the extended signal and appropriate IMFs are selected for reconstruction when the extension part is truncated. The implementation process is shown in Figure 1.

#### 2.3.1. Signal Extension Based on Support Vector Regression

Support vector regression is a “tolerant” regression model, which maps the data *x* ∈ *R* ^*n*^ to a high-dimensional feature space *H* through a nonlinear mapping function *φ* and performs the linear regression in this space correspondingly [21]. It can be abstracted into the following expression:

(3) $$\begin{array}{c} \left\{\begin{array}{c} \left(x\right)=w\cdot \varphi \left(x\right)+b,\\ \varphi :x⟶H, \end{array}\right. \end{array}$$

where *w* is the normal vector of the regression hyperplane and *b* is the threshold. Based on this algorithm, we extend the time series by steps (1) to (4):

1. Construct a training set *T* = {(*x*_1_, *y*_1_),…, (*x*_*n*_, *y*_*n*_)} using the left-end data of the time series
2. Select precision parameter “*ε*”, error penalty factor “*C*”, loss function “*e*,” and kernel function *k*(*x*_*i*_, *x*_*j*_) to construct the SVR model

(4) $$\begin{array}{c} f\left(x\right)=\overset{n}{\underset{i=1}{\sum }}\left(a_{i}^{*}-a_{i}\right)k\left(x_{i},x\right)+\bar{b}, \end{array}$$

  where *a*_*i*_^*∗*^, *a*_*i*_ *i*=1,2,…, *n* are Lagrange multipliers and only a small part which corresponds to the so-called support vector (SV) is not zero
  In this step, the SMO [22] algorithm, the key point of which is to decompose a complex optimization problem into several suboptimization problems that are often easy to solve, is performed by iteratively selecting subsets of {*a*_*i*_^*∗*^, *a*_*i*_} only of size 2, leaving all the other kept fixed and optimizing the suboptimization problems of equation (5) until a set of {*a*_*i*_^*∗*^, *a*_*i*_} is optimized:

(5) $$\begin{array}{c} \underset{0\leq a_{i}^{*},a_{i}\leq C}{\min}\left(\frac{1}{2}\overset{n}{\underset{i,j=1}{\sum }}\left(a_{i}^{*}-a_{i}\right)\left(a_{j}^{*}-a_{j}\right)k\left(x_{i},x_{j}\right)+\varepsilon \overset{n}{\underset{i=1}{\sum }}\left(a_{i}^{*}+a_{i}\right)-\overset{n}{\underset{i=1}{\sum }}y_{i}\left(a_{i}^{*}-a_{i}\right)\right). \end{array}$$

  The threshold $\bar{b}$ is derived by

(6) $$\begin{array}{c} \frac{1}{N_{\text{SV}}}\left\{\underset{0<a_{i}<C}{\sum }\left[y_{i}-\underset{x_{j}\in \text{SV}}{\sum }\left(a_{j}^{*}-a_{j}\right)k\left(x_{j},x_{i}\right)+\varepsilon \right]+\underset{0<a_{i}^{*}<C}{\sum }\left[y_{i}-\underset{x_{j}\in \text{SV}}{\sum }\left(a_{j}^{*}-a_{j}\right)k\left(x_{j},x_{i}\right)-\varepsilon \right]\right\}, \end{array}$$

  where *N*_SV_ is the number of support vector samples
(3) Use the trained SVR model to extend a finite number of maxima and minima points to the left end of the time series
(4) By repeating steps (1)–(3) for the right end data, we can get the left and right ends of the time series being extended

Considering that SVR model is to seek a linear regression function to fit all the samples to minimize the total variance of the sample from the hyperplane, we let *C* equal to infinity and *ε* be zero to improve the regression accuracy. Furthermore, we have also used the commonly used *ε*-insensitivity loss function and linear kernel function for the sake of convenience.

#### 2.3.2. Signal Decomposition and Reconstruction Based on EEMD

In the first step of EEMD, an independent identically distributed and zero mean white noise whose intensity (*N*_p_) should match the noise intensity in the signal as much as possible is added and then EMD is applied to drive a set of IMFs. These steps are repeated for *N* times to conclude an ensemble of IMF sets and, finally, the ensemble should be averaged to receive one set of IMFs.

EMD of the main work is performed basically by a sifting process as follows:

1. Assign the original signal to *y*(*t*).
2. Find the local maxima and minima of the signal *y*(*t*).
3. Interpolate (cubic spline interpolation here) between the local maxima and minima to generate upper and lower envelops: *e*_max_(*t*) and *e*_min_(*t*).
4. Subtract the mean value of envelops from *y*(*t*)

(7) $$\begin{array}{c} c\left(t\right)=y\left(t\right)-\frac{\left(e_{\max}\left(t\right)+e_{\min}\left(t\right)\right)}{2}. \end{array}$$

(5) Calculate the sift relative tolerance (*rtol*), the stop criterion of IMF, which is set to 0.2 in this paper

(8) $$\begin{array}{c} rtol=\frac{{\left(c_{i-1}\left(t\right)-c_{i}\left(t\right)\right)}^{2}}{c_{i}{\left(t\right)}^{2}}, \end{array}$$

  where *c*_*i*_(*t*) and *c*_*i*−1_(*t*) denote the current and previous *c*(*t*), respectively.
(6) Determine if *rtol* is less than 0.2, and if so, terminate the loop and treat the current *c*(*t*) as an IMF; otherwise assign *c*(*t*) to *y*(*t*) and continue iterating the steps from (2) to (6)
(7) Subtract *c*(*t*) from the original signal and repeat the steps from (1) to (7) until *y*(*t*) can never be decomposed, then the original signal can be expressed as

(9) $$\begin{array}{c} y\left(t\right)=\overset{n}{\underset{i=1}{\sum }}{\text{IMF}}_{i}\left(t\right)+r\left(t\right), \end{array}$$

where *n* is the total number of IMFs and *r*(*t*) is the residual component

Typically, the original signal will be decomposed into several IMF components, and the first few correspond to the high-frequency band of the time series and the last few correspond to the low-frequency band. As a result, we can obtain the denoised signal by selecting the target IMFs for reconstruction based on the signal and noise frequency distribution characteristics; that is to say, if the noise frequency is in high-frequency band or higher than the signal frequency, we can zero the first few IMFs and reserve the other IMFs where the signal is located and vice versa.

## 3. Results and Discussion

To demonstrate the denoising performance of the proposed SVR-EEMD method, we applied it to the simulated data and real-world PPG data. For the simulated data, we use SNR and correlation coefficient (Corr) to evaluate the effectiveness of this method and select precision rate (*P*) and recall rate (*R*) of the pulse wave peak as estimations of this method on the real-world PPG data.

### 3.1. Experiments for the Simulated Data

**Figure 2:**
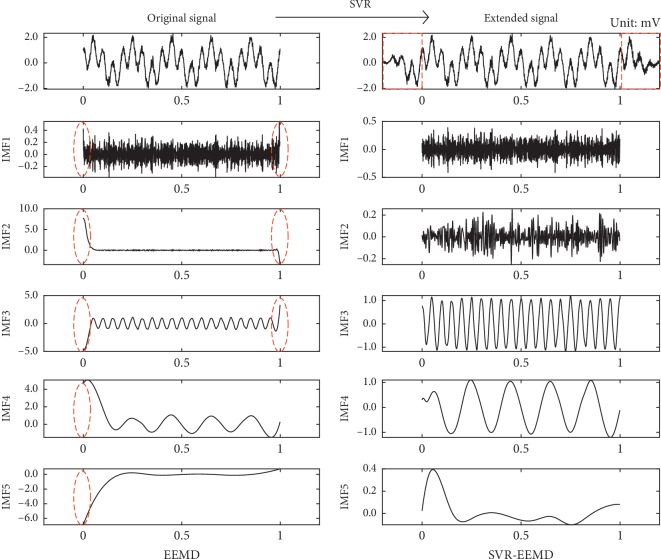
IMFs of the original signal decomposed by EEMD (left) and IMFs decomposed by SVR-EEMD (right). The red rectangular areas are the extension part of the original signal, and the elliptical areas identify the “end effect.”

We choose *N* = 100 and *N*_p_ = 0.0316 to make sure that the EEMD and SVR-EEMD methods are under the same decomposition condition.  Figure 2 depicts the IMF components of the simulated signal decomposed by those two methods in detail.

In Figure 2, we can discover that, first, unlike the general mirror extension or zero-padding operation, our SVR model can predict the previous and future trend of the signal and extend it accurately. Second, the IMFs are arranged in order of frequency from high to low and in this decomposition, IMF^3rd^ and IMF^4th^ correspond to the signal components of the simulated signal with frequencies of 20 Hz and 5 Hz, respectively, while the other corresponds to the noise components. Third, all the IMFs (left) decomposed by EEMD have different degrees of divergence at the left and right ends, especially the left. In contrast, the SVR-EEMD method suppresses this effect to a large extent.

**Figure 3:**
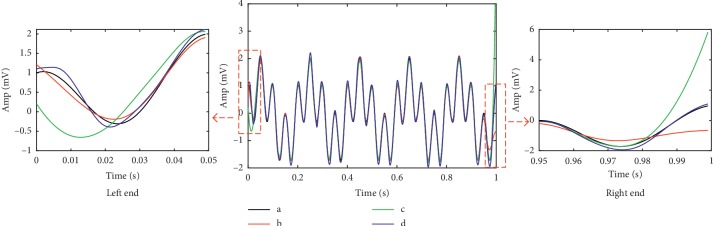
Comparison of the processed signals and original signal. (a) Original signal. (b) FIR low-pass filter. (c) EEMD. (d) SVR-EEMD. The left and right subgraphs depict a detailed comparison of the left and right ends within 0.05 s of the processed signals, respectively.

Figure 3 compares the processed signals by FIR low-pass filter (cutoff frequency at 22 Hz), EEMD, and SVR-EEMD method. Due to the “end effect,” the signal reconstructed by EEMD has severe distortion at both sides and the filtered data also have small deviation from the original signal because of phase shift, in which circumstances only the signal reconstructed by SVR-EEMD maintains a high degree of consistency with the original signal as the left and right subgraphs show. We calculated the SNR and Corr listed in Table 1, which proves the SVR-EEMD an effective method to significantly suppress the “end effect” and filter out noise in the signal.

### 3.2. Experiments for the Real-World PPG Data

**Figure 4:**
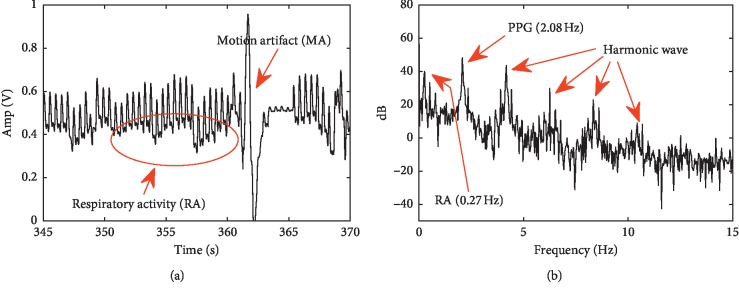
Time (a) and frequency (b) domain distribution characteristics of the PPG signal of patient 25.

**Figure 5:**
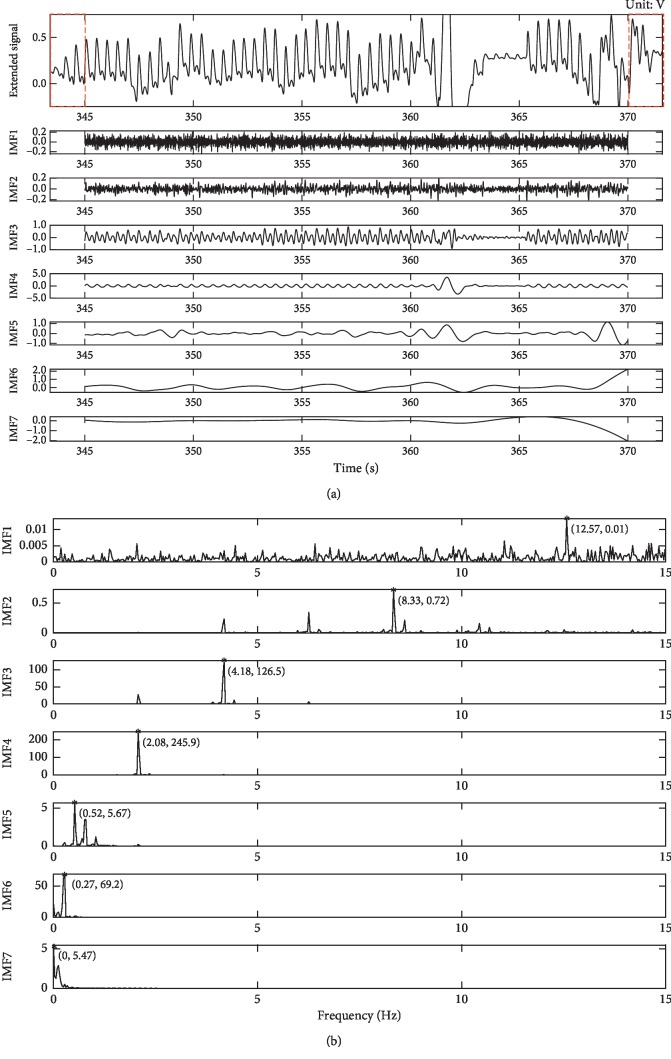
IMFs and PSD of PPG signal decomposed by SVR-EEMD. (a) Time series of IMFs. The first row is the extended version of the PPG signal shown in Figure 4(a). (b) PSD of each IMF. The corresponding maximum frequency point is marked with a red asterisk and values in parentheses.

Figures 4(a) and 4(b) briefly describe the time-frequency distribution of the PPG signal of the patient 25 during the 345–370 s period. We can see that there was a strong motion disturbance (red arrow) around the 362 s and the PPG signal was completely submerged in the noise. In addition, we can clearly see that the respiratory activity (red elliptical area) is superimposed on the PPG signal, which is also confirmed in Figure 4(b). In Figure 4(b), the respiratory rate is about 0.27 Hz consistent with the dataset record and the signal also contains a large number of harmonics in addition to PPG signal (about 2.08 Hz). We use the proposed method (*N* = 30, *N*_p_ = 0.6) to decompose the data, and results are shown in Figure 5(a). Additionally, we draw the power spectral density (PSD) map of each IMF in Figure 5(b).

**Figure 6:**
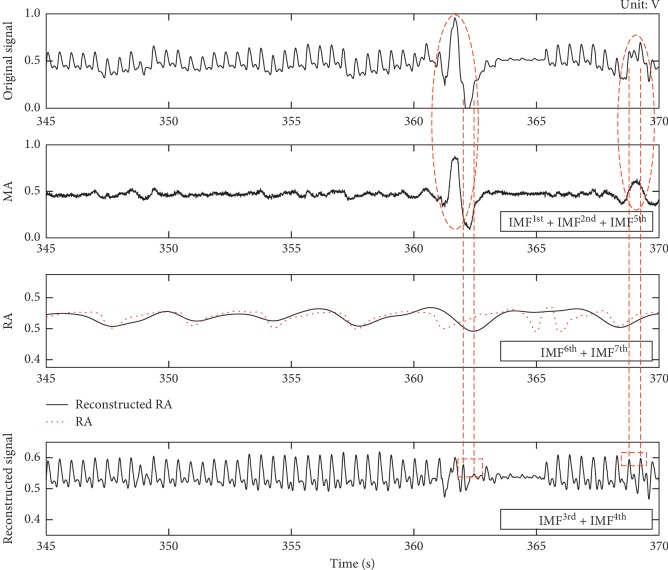
MA, RA, and PPG signals reconstructed by the SVR-EEMD method. The red elliptical areas are two ripples decomposed from the original signal, and the red dotted curve presents the recorded respiratory activity in the dataset. The two red rectangles indicate the recovered peaks of the reconstructed PPG signal by IMF^3rd^ plus IMF^4th^.

It can be seen from Figure 5(a) that the left and right ends of the original PPG signal are accurately extended by three peaks (red rectangular area) after the SVR extension, and the extended signal is decomposed into seven IMF components in different frequency bands by EEMD. Among the IMFs, there is no divergence at each component, which proves that the SVR-EEMD method can solve the “end effect” problem in EMD when decomposing PPG signals. From the perspective of IMF frequency, IMF^1st^ and IMF^2nd^ are mainly random noise and harmonics with relatively higher frequency and lower intensity compared with IMF^3rd^ and IMF^4th^ (the maximum intensities of IMF^1st^ and IMF^2nd^ are 0.01 and 0.72 with corresponding frequencies of 12.57 Hz and 8.33 Hz, respectively, while the maximum intensities of IMF^3rd^ and IMF^4th^ are 126.5 and 245.9 with corresponding frequencies of 4.18 Hz and 2.08 Hz, respectively). IMF^4th^ is the peak position of PPG signal whose details can be found in IMF^3rd^. IMF^6th^ and IMF^7th^ are the least lower frequency bands corresponding to the respiratory activity elliptically annotated in Figure 4(a), and the frequency of IMF^5th^ is the most mixed with two distinct ripples at 362 s and 369 s. We reconstructed the PPG signal, respiratory signal, and interference signal shown in Figure 6 with these IMFs. It can be found that the two evident motion artifacts (red elliptical area) in the original signal have been decomposed into the MA signal, and the reconstructed RA signal (black solid curve) is also in good agreement with the respiratory activity (red dotted curve) recorded in the dataset. Compared with the original signal, the reconstructed PPG signal not only filters out most of the interference but also successfully recovers the PPG signal (red rectangular area) that is submerged in MA noise. However, at the 362 s moment, the interference is too strong to recover the PPG signal clearly but enough to detect the PPG peak position.

We count the ratio of the number of successfully recognized peaks to the total number recognized as the precision rate and to the actual number in PPG data as the recall rate to verify the performance of the FIR filter (cut-off frequency at 12 Hz according to PPG signal frequency range), EEMD, and SVR-EEMD method again using the patient data, and the results are listed in Table 2.

It can be found statistically from Table 2 that the EEMD method is slightly better than the FIR filter in terms of precision and recall. For data of patients 2, 5, 34, 38, 43, and 50, the EEMD method works better than the FIR filter, while for patients 33 and 45, the FIR filter does indeed better than the EEMD method. Unsurprisingly, the SVR-EEMD method is more often outstanding than the previous two methods. The reasons we analyzed for this result may be that first, the frequency components in PPG signals of different patients are different, especially those whose pulse rate is extremely unstable, resulting in different methods with different treatment results; second, the “end effect” causes the signal to diverge during decomposition and leads to false peaks or missing peaks, to make matters worse, and this divergence may penetrate into the signal and contaminate the entire data sequence. Third, the random nature of the auxiliary added Gaussian white noise may cause large fluctuations at a certain position of the signal, which could make the effectiveness of the EEMD method not as effective as the FIR filter. Furthermore, we calculated the correlation coefficient and mean delay time (MDT) of the data processed by those three methods as shown in Table 3.

The mean delay time is calculated by equation (10), where *n* is the total number of peaks for successful recognition at *t*_Eig_ in the processed data and *t*_Eig_′ is the time at which the peaks of the original data are located is defined as the average of the sum of the absolute time difference between the successfully identified peaks and the corresponding peaks in the original data:

(10) $$\begin{array}{c} \text{MDT}=\frac{\sum_{i=1}^{n}\text{abs}\left(t_{\text{Eig}}-t_{\text{Eig}}^{\prime }\right)}{n}. \end{array}$$

We can see that, due to the phase shift effect, the FIR filtered data have a significant time delay phenomenon, thus with a relatively lower correlation coefficient. Inversely, the EEMD and SVR-EEMD methods have higher correlation coefficients while achieving lower latency. Moreover, the SVR-EEMD method solves the “end effect” problem and improves both the MDT and Corr indicators.

### 3.3. Discussion

**Figure 7:**
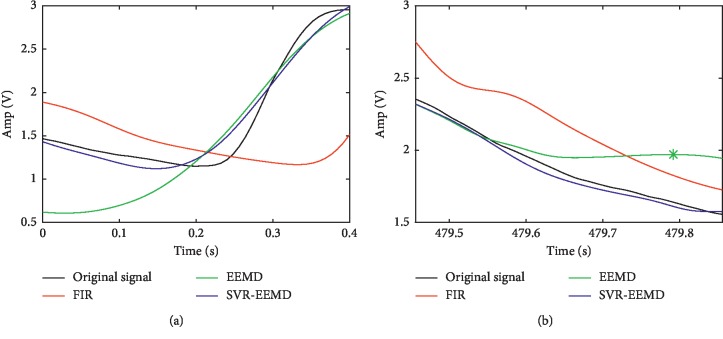
End comparison of original signal and signals processed by different methods. (a) Left end. (b) Right end.

In Table 2, the EEMD method is generally better than the FIR filter, except for patients 33 and 45. Taking the data of patient 45, Figures 7(a) and 7(b), respectively, describe in detail the comparison of the left and right ends between the processed data and the original data.

It can be seen that the data filtered by the FIR filter have a serious phase delay problem, which is the reason why the MDT is longer and the Corr is lower in Table 3. The left and right ends of the data reconstructed by the EEMD method also have different degrees of divergence and deviate from the original data trend. Even worse, a false peak appears in the right end data, which reduces the precision and recall of the EEMD method to some extent. In contrast, the SVR-EEMD method does not have these two problems and has achieved good results.

**Figure 8:**
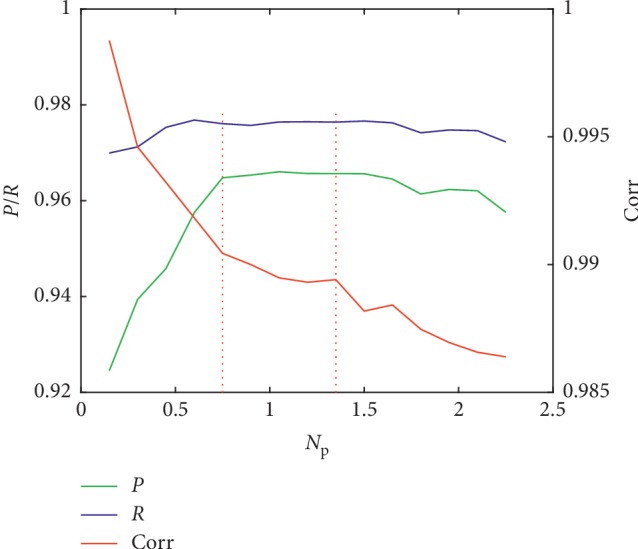
Average of Corr, *P*, and *R* when different noise intensities are superimposed on the PPG signal.

In addition, the intensity of noise superimposed on the signal has an important influence on the decomposition effect of the EEMD method. For the simulated signal, we can calculate the relative energy of noise and select appropriate noise intensity. However, for the real PPG data, we have no prior knowledge of the noise in the data, but we can estimate the noise intensity distribution range by posterior statistics.  Figure 8 shows the averaged correlation coefficient, precision, and recall of the 10 sets of data processed by the SVR-EEMD method applying different noise intensities ranging from 0.15 to 2.25 and the suitable noise intensity range is 0.75–1.25, the key point of which is that how much it should be applied needs further study.

Although the phase shift characteristic of the FIR filter makes the filtered data less correlated with the original data, the filter is simpler and easier to use. If a low phase shift or zero phase shift filter is used, the result will be improved, but the signal and noise in the data cannot be decomposed into different intrinsic mode functions like the EEMD method does.

## 4. Conclusions

In order to solve the “end effect” problem in the EEMD method, this paper proposes an SVR-EEMD method based on support vector regression extension and applies it to the denoising of PPG signals. Both simulated data and real-world PPG data are used to compare the denoising performance of the FIR low-pass filter, EEMD, and SVR-EEMD methods. For the simulated data, the SNR of which processed by the SVR-EEMD method improves nearly three times higher with a correlation coefficient over 0.99. For the real-world PPG data processed by the SVR-EEMD method, not only the precision and recall are higher than the other two methods but also it maintains high consistency with the original PPG data. The results of the simulated data and real-world PPG data prove that the proposed method can overcome the “end effect” problem of the traditional EEMD method in decomposition, which can improve the decomposition performance and bring beneficial results for nonlinear and nonstationary signal analysis.

## Figures and Tables

**Table 1:** SNR and Corr of the processed signals.

Method	SNR	Corr
FIR	37.83	0.9886
EEMD	18.35	0.9203
SVR-EEMD	44.54	0.9978

**Table 2:** *P* and *R* when PPG signals are dealt with different methods.

Num	Peaks	P			R		
		FIR	EEMD	SVR-EEMD	FIR	EEMD	SVR-EEMD
2	454	0.960	0.980	0.981	0.998	0.989	1.000
5	524	0.994	0.996	1.000	0.985	0.985	0.996
33	633	0.962	0.957	0.965	0.964	0.959	0.967
34	219	0.991	0.995	0.995	0.991	0.986	0.995
37	645	0.994	0.994	0.995	0.989	0.989	0.998
38	641	0.954	0.958	0.964	0.969	0.964	0.969
43	717	0.986	0.993	0.996	0.983	0.991	0.997
45	526	0.945	0.936	0.947	0.941	0.939	0.949
50	293	0.983	0.986	0.993	0.973	0.997	1.000
53	626	0.957	0.957	0.963	0.957	0.958	0.963

**Table 3:** Corr and MDT when PPG signals are dealt with different methods.

Num	MDT(s)			Corr		
	FIR	EEMD	SVR-EEMD	FIR	EEMD	SVR-EEMD
2	0.121	0.012	0.008	0.337	0.968	0.977
5	0.126	0.010	0.009	0.342	0.997	0.998
33	0.218	0.010	0.008	0.013	0.982	0.999
34	0.123	0.010	0.009	0.114	0.969	0.995
37	0.118	0.014	0.008	0.336	0.984	0.987
38	0.070	0.002	0.002	0.488	0.988	0.991
43	0.145	0.022	0.019	0.321	0.656	0.916
45	0.117	0.052	0.049	0.442	0.991	0.993
50	0.160	0.026	0.022	0.146	0.977	0.991
53	0.133	0.011	0.005	0.301	0.986	0.991

## Data Availability

The simulated data used to support the simulation part of this study are available from the corresponding author upon request, and the real-world PPG data can be obtained from BIDMC PPG and Respiration Dataset of Physio Bank at https://www.physionet.org/physiobank/database/bidmc.
